# Epidemic Cerebro-Spinal Meningitis

**Published:** 1873-05

**Authors:** S. Hudson

**Affiliations:** Medina, Ohio


ARTICLE II.—*Epidemic Cerebro-Spinal Meningitis.* Extracts from an Essay read before the Medical Association of Northeastern Ohio, by S. HUDSON, M.D., of Medina, Ohio.

* * * * The disease generally prevails in the winter or colder months of the year, showing that it is not caused by malaria; indeed, I have never known it to prevail in miasmatic or malarious districts, as an epidemic. Whether it is contagious or not is not definitely settled, much difference of opinion existing upon this point.

As to its pathology, I believe it is admitted by all to be an inflammation of the meninges of the brain and spinal cord; and this inflammation varies from the highest, or sthenic, to the lowest, or asthenic, type; both of these conditions obtaining sometimes—at different periods of the disease—in the same person. Such was the character of most of the cases in the epidemic that occurred in Medina, Ohio, during the winter of 1863–4, the mortality of which was about 80 per cent.

I come now to the most interesting question connected with the consideration of this disease, *i. e.:* What is it—what is its true nature? I answer, that after close observation and treatment of many cases, I have had the conclusion forced upon my mind that *epidemic cerebro-spinal meningitis* and *epidemic erysipelas* are one and the same disease. In other words, that cerebro-spinal meningitis is nothing more than an erysipelatous inflammation of the meninges of the brain and spinal cord. My reasons for this opinion will appear before I close, and it is for you to judge if they be valid.

The symptoms of the disease vary much in different cases. Sometimes the attack is so sudden that the patient is found insensible, and so continues until death; but as a general thing several days' indisposition, accompanied by cephalalgia, precede the full development of the malady, which is ushered in by a chill of more or less intensity and duration. I have known these chills to vary from slight chilliness to an attack so severe that the patient succumbed without reaction ever taking place. Reaction, however, usually occurs; febrile symptoms manifest themselves, and you shortly after discover that your patient has hemiplegia; or is blind; or perhaps deaf; speechless, with contracted pupils; opisthotonos, or convulsions, may follow in rapid succession, and of the most alarming character; tongue covered with a moist, white coating; bowels usually constipated; incessant vomiting. Retention of urine is very frequent, as is also dysphagia. In other cases the patient complains only of headache; is inclined to sleep most of the time, the drowsiness finally passing into coma with stertorous breathing, in which condition he dies. In the cases which came under my notice, the most of them had, at the onset, a peculiar lazy, sluggish character of pulse, more easily recognized than described. In many of the cases I was enabled to make my diagnosis at once from the peculiar condition of the pulse. In the most malignant cases the patients would not survive the attack more than thirty-six to forty-eight hours, though some would linger four or five days; when they lived longer than this they would sometimes last as many weeks. Two of apparently the most severe cases we had lingered between death and life for some six months, and both finally recovered.

I would now invite your attention to a brief description of an epidemic that visited our place in the winter of 1863–4. Early in the winter an epidemic of erysipelas made its appearance. Many died, for it was phlegmonous erysipelas prevailing as a malignant epidemic. In some cases the patient would be taken with a pain in the end of a finger or toe, and die within forty-eight hours without any external appearance of the erysipelas. In others the disease would affect the bowels, brain, or spinal cord, and the patient would die in a few hours. During the prevalence of this epidemic I was shocked to learn that one of our county officials was thought to be dying, and of a new disease. Through the kindness of friends I was invited to see him. His physician had diagnosticated congestive chill. I found him *in articulo mortis.* From his appearance and the history of the case, my diagnosis was cerebro-spinal meningitis. His body was slightly covered with petechiæ, facial muscles contracted, head thrown back, and pupils dilated. Duration of the disease, forty-eight hours. Following this case came others, thick and fast, of as great or lesser intensity, but of the same general character. I will relate the prominent points of a few of them.

Case I. Mrs. B. Taken with a chill Feb. 4, 1864, which lasted twelve hours. As soon as she reacted from the chill her respirations went up to fifty per minute, while the pulse was heavy and sluggish at forty to the minute. She soon became insensible and went into a deep, heavy sleep, from which she could with difficulty be aroused. This state continued until about noon the next day, being the fifth from the attack, when she partially aroused and would answer questions, though not always correctly. Now her pulse became more rapid, her breathing less so; her stomach would tolerate nothing, and the characteristic pea-green vomit occurred. These symptoms continued during the day, with bowels constipated, retention of urine, some opisthotonos, pupils contracted, and partial hemiplegia of the left side, her body slightly covered with petechiæ. Consequently I diagnosed spotted fever, or cerebro-spinal meningitis, and so informed her friends. There was no abatement of the severity of the symptoms until the morning of the seventh day, when I found her apparently much better. The nurse pulled down the bed clothing, and said, "Here, doctor, is your spotted fever, or cerebro-what-do-you-call-it!" And sure enough, what did I call it? A more well defined and plainly marked case of phlegmonous erysipelas I never saw. The lady in attendance said: "Doctor, I thought *you* had seen enough of erysipelas to know it from spotted fever." The neck of the patient, both back and front, as well as the anterior portion of her chest, was completely covered with an erysipelatous blush, which was persistent for seven days, when it was followed by desquamation of the cuticle, but by no cellular pus formation, of which I had grave apprehensions. After a lingering illness the patient recovered. I had here primarily as well marked a case of cerebro-spinal meningitis as I ever saw (and I have treated a great many cases of it); her symptoms were almost identical with those of others who were at the same time suffering from the disease and dying almost daily.

Case II. The next case was that of Miss R., æt. 21, living but a few steps distant from the house of case I. She too had rigors, which lasted in her case some fifteen hours.

Reaction came on slowly, followed by vomiting, as in the other case; pulse slow and heavy; respiration very rapid; stomach would retain nothing. Hemiplegia of left side on the second day; she was deaf, blind, and speechless; pupils contracted; bowels constipated, and the urine retained. Diagnosis: spotted fever. (This was before my other case "went back on me," as they were attacked at nearly the same time.) As no external erysipelas appeared in this case, my diagnosis stood approved. After many weeks this case also recovered.

Cases III and IV. In the family of Miss R—— two younger brothers were taken down within a few hours of their sister's attack, both of whom had phlegmonous erysipelas of scalp and face, and recovered in three or four weeks. * * * * *

I might multiply cases, but suffice it to say that parallel ones to those cited were numerous, not only in our own village, but all through the adjoining townships. It was no uncommon occurrence for one member of a family to have cerebro-spinal meningitis and in less than twenty-four hours another would be taken down with erysipelas. In other cases the disease would commence as cerebro-spinal meningitis and terminate as erysipelas, (as in the case of Mrs. B——); in still another class a case of erysipelas of face and scalp would be progressing favorably, when suddenly the eruption would fade, and the patient would die with all the symptoms of spotted fever. In others yet, the attack would be upon the extremities, and so severe would be the pain that it was almost impossible to mitigate it; this would continue from twenty-four to forty-eight hours, when metastasis to the brain or spinal cord would occur, and the patient succumb within a few hours.

Those of my medical brethren who have passed through one or more of those terrible epidemics of erysipelas, which have visited Northern Ohio at several times and in numerous places since 1842, will doubtless call to mind many such cases as I have described.

These cases are of importance, as I believe it is admitted by all writers on epidemic erysipelas, that there are scarcely any tissues of the body exempt from its invasion; that the meninges of the brain and spinal cord, the thoracic and abdominal viscera, skin, muscles, nerves and bones, may any or all of them be the seat of this disease.

Another point favoring the idea of the identity of cerebro-spinal meningitis and epidemic erysipelas, is their frequent simultaneous appearance and identical symptoms of invasion. In 1864, after being deceived a number of times, I was more cautious in my diagnois, for I could not tell, at the onset of the disease, whether it would show itself externally or not. As spring came on, the disease relaxed in severity, but up to the last cases preserved its erratic course as to external manifestations.

From 1864 to 1871 we were free from a visitation from this scourge, but Dec. 9, 1870, it reappeared, selecting as its first victim a little son of my own, aged eight years. He had pain in his ankle. of unusual severity, following a slight chill. I suspected rheumatism, and so treated him for a short time, but on testing his urine and finding no excess of uric acid, and the excruciating pain continuing despite my opiates, I called in my friend Dr. Hard, but we were unable to make a satisfactory diagnosis. The 4th, 5th and 6th days the pain increased in severity until the poor child begged us to kill him and end his suffering. The leg now for the first time was slightly swollen, and the least jar would almost throw him into convulsions; bowels constipated, and urine scanty; pulse nearly normal. On the morning of the 7th day I saw to my sorrow that the meninges of the brain and cord were affected; then followed in rapid succession the symptoms usually attending meningitis, and in about twenty-four hours he expired.

The next case in this epidemic was that of Mrs. W——, æt. 35, living but a few doors from my house, and attacked shortly after my boy. Pain in the ankle, identical in character and severity with that of my son, until the third day, when her foot and leg took on an erysipelatous inflammation which lasted eight or ten days, when she slowly recovered. This was a well defined case of phlegmonous erysipelas, but had the disease invaded the cerebro-spinal meninges, as it did in the preceding case, instead of coming out on the leg, it would have been recorded as one of spotted fever.

I have not time to make special report of all the interesting cases that fell under my observation last winter, (1871); they were numerous, and of the same general character as those of the epidemic of 1864, and Dr. Hard and myself had the misfortune to treat nearly all of them.

Our cases of pneumonia, several of them after becoming convalescent, suddenly went off with brain trouble, with all the symptoms of spotted fever. This was the case in both epidemics spoken of; and our measles patients fared but little better. Indeed there seemed to be a tendency to brain trouble in every form of disease with which we had to contend. * * * *

